# Epidemiology of Periodontal Diseases in Individuals With Diabetes in Saudi Arabia: A Systematic Review

**DOI:** 10.7759/cureus.67721

**Published:** 2024-08-25

**Authors:** Abdulaziz A AlJaafary, Raed Alahmari, Nora A AlHumaid

**Affiliations:** 1 Family Medicine, King Fahad Medical City in Riyadh, Health Cluster 2, Ministry of Health, Riyadh, SAU; 2 Family Medicine, King Fahad Medical City, Riyadh, SAU; 3 Medicine, King Fahad Medical City, Health Cluster 2, Ministry of Health, Riyadh, SAU

**Keywords:** periodontitis, gingivitis, periodontal diseases, diabetes mellitus type 2, type 1 diabetes mellitus (t1d)

## Abstract

Diabetes mellitus (DM) is one of the most common and prevalent metabolic disorders worldwide. It affects men, women, and children and can cause severe organ damage, including kidney damage. The two most common types of DM are Type 1 DM and Type 2 DM. Similarly, periodontal disease (PD) affects a significant portion of the population and involves the tissues supporting the teeth. The bidirectional relationship between diabetes and PD is a well-established phenomenon. However, no comprehensive study on the prevalence of PD among diabetic patients has been conducted on the Saudi population. This systematic review aims to examine the current literature on the impact of these conditions on the Saudi population. A literature search was conducted from January 2001 through June 2024 using Scopus, PubMed, and Google Scholar. An extensive search was performed, duplicates were removed, and inclusion and exclusion criteria were applied. Nine studies were selected for review, adhering to Preferred Reporting Items for Systematic Reviews and Meta-Analyses (PRISMA) guidelines. The retrieved studies confirm the relationship between DM and PDs in regional studies. The review highlights the need for integrated healthcare approaches to manage both conditions effectively. Further research is recommended to understand the causal mechanisms and develop comprehensive treatment strategies.

## Introduction and background

Diabetes mellitus (DM) is a metabolic disorder that can affect men, women, and children, and it is one of the most prevalent diseases around the globe. It is characterized by the inability of beta-pancreatic cells to produce insulin. There are two main types of DM: Type 1 and Type 2. Type 2 DM (T2DM) is most common in adults, characterized by either efficient insulin production with the body becoming resistant to insulin or inefficient insulin production. Type 1 DM (T1DM), a relatively early-onset disease during childhood, is characterized by little or no insulin production from the pancreas [1]. Insufficient insulin production or insulin resistance can lead to an imbalance of glucose levels in the blood, ultimately leading to defects in the metabolism of fats, proteins, and carbohydrates [2].

Diabetes is one of the most prevalent diseases in the United States, with over 21 million Americans suffering from it. It is a major burden on the healthcare system and is associated with higher mortality and morbidity ratios among patients [3]. Similarly, according to reports published by the World Health Organization (WHO), the Kingdom of Saudi Arabia has the second highest prevalence of diabetes in the Middle East and ranks seventh globally. The country currently has around 7 million diagnosed diabetic and 3 million pre-diabetic patients [4]. The constant increase in the incidence of DM is a significant burden on the healthcare system due to its high mortality and morbidity ratios.

Periodontal diseases (PDs) are a group of conditions affecting the gum tissues responsible for holding the teeth, potentially leading to poor attachment or tooth loss. The disease has multiple causes, including bacterial pathogenesis, poor dental hygiene, smoking, and a weakened immune system. It is also caused by familial factors, such as gingivitis, which leads to periodontitis [5]. The condition is not only linked to these factors but is also associated with diabetes, complications related to pregnancy, heart disease, hypertension, and lung disorders [6, 7].

Among other conditions, the most important factor studied extensively is its relationship with DM. It has been observed that the glycemic index plays a major role in its management and progression, and high hemoglobin A1c (HbA1c) has been linked with poor oral health [8]. The first report suggesting the link between PD and DM was published in 1960. Since then, many human and animal studies have confirmed the relationship between the two conditions. Not only are these two conditions related, but diabetes also impacts treatment regimens, making it difficult to treat periodontal illness in many cases [9, 10]. The relationship is not unidirectional but rather bidirectional, as diabetes increases the risk of developing PDs, and a higher incidence of periodontitis can also lead to the progression of diabetes [9]. The possible explanation for the higher prevalence of PD in diabetes is that it may alter the subgingival bacterial community, supporting an environment that can enhance the ability of pathogens to grow easily [11]. It has also been observed that systemic inflammation markers like IL-6, TNF-α, and C-reactive protein increase in PD [12]. Numerous studies support this association by demonstrating that people with diabetes have a higher incidence of periodontitis than those without the illness. Further highlighting the reciprocal association between diabetes and PD, periodontal care has been shown to lower inflammatory markers in diabetic patients [13].

There are many reports that suggest the connection between periodontal disorders and DM. Many reports from different geographical locations are already present in the literature; however, very few studies from the Saudi population focus on the prevalence of PD in diabetic patients [14]. The current review aims to collect comprehensive information on the incidence of periodontal illnesses in people with diabetes and the relationship between diabetes and PDs to investigate the reciprocal nature of these two ailments.

## Review

Materials and methods

This systematic review aimed at evaluating the relationship between PD and DM in the Saudi population. We systematically reviewed epidemiological, cross-sectional, retrospective, and observational studies published from 2000 to June 2024 to assess the association between PD and DM. Although considerable data exist on the relationship between DM and PD, only a few studies have reported the prevalence in the Saudi population. We searched multiple electronic databases, including Google Scholar, Scopus, and PubMed, using keywords such as "Periodontal Diseases" and related MeSH terms like "Gingivitis" and "Periodontitis," along with "Diabetes Mellitus" and associated terms, combined with "Saudi Arabia" using Boolean operators AND and OR as appropriate. The inclusion criteria encompassed studies linking PDs with diabetes conducted within Saudi Arabia, where full-length articles were available and written in English. Studies conducted in other countries or in languages other than English were excluded. After a thorough screening, we eliminated 8,000 duplicate articles and excluded studies outside the specified timeline.

**Figure 1:**
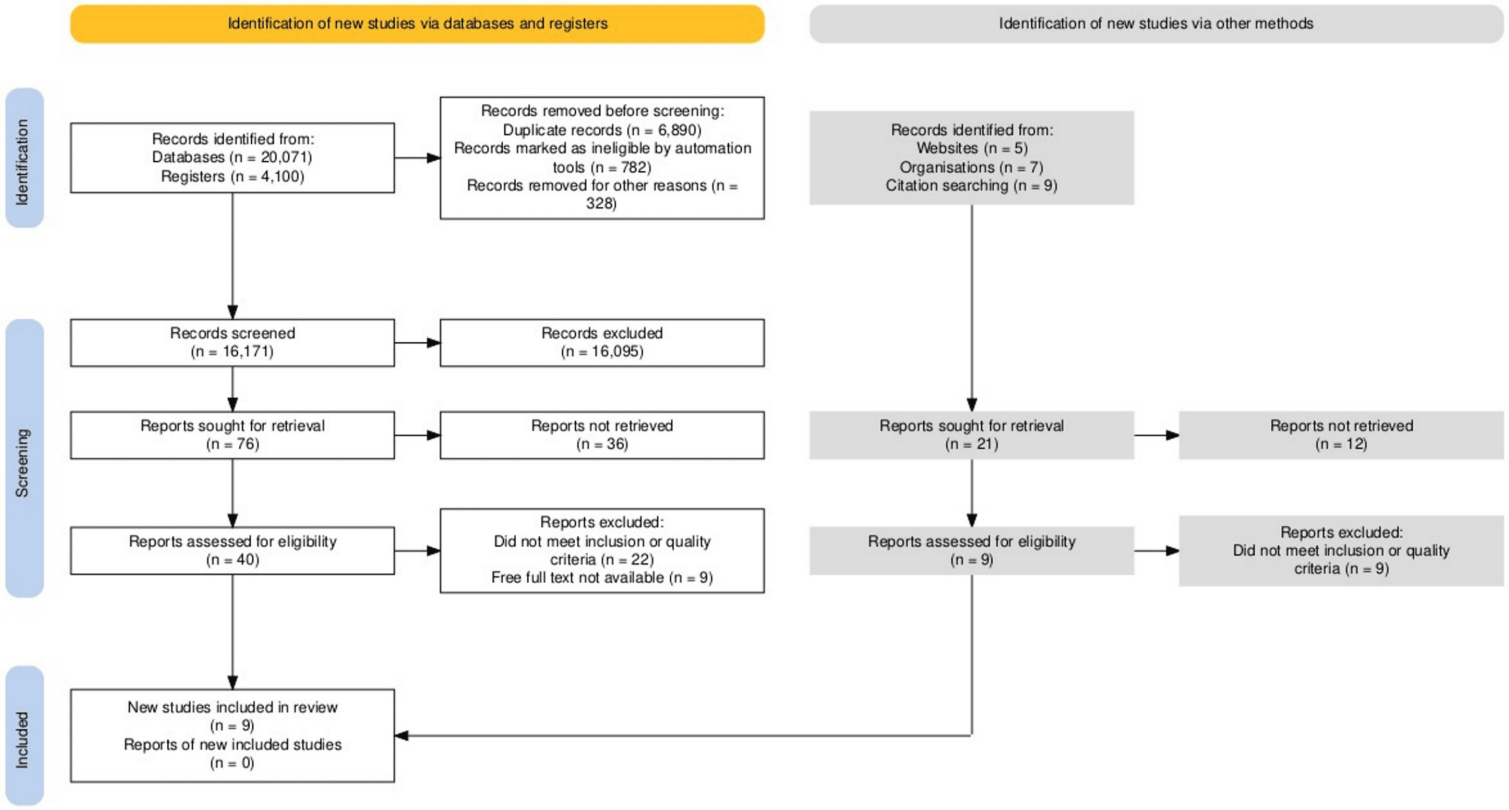
PRISMA flowchart depicting the study selection process

Furthermore, we excluded 31 articles due to insufficient details, non-original research, language differences, and lack of proper evidence. No articles were excluded based on the nature of their findings. Ultimately, nine articles were reviewed, following the 2020 Preferred Reporting Items for Systematic Reviews and Meta-Analyses (PRISMA) guidelines (Figure 1) [15]. The risk of bias analysis was conducted using the ROBINS-E tool [16], and the results were visualized using the Robvis tool (https://mcguinlu.shinyapps.io/robvis/).

Results

The characteristics and study designs of the included studies are outlined in Table 1. Most of the studies on PDs and DM were conducted in Riyadh, the capital of Saudi Arabia. The majority were case-control studies, including those involving diabetic and nondiabetic individuals. Three cross-sectional studies and one descriptive retrospective study were also included in the retrieved studies. A total of 3,773 participants were included, of which 1,214 were diabetic and nondiabetic individuals. The characteristics of the study participants, including diabetes and gingivitis status, mean age, and gender, are detailed in Table 2.

**Table 1:** Selected studies with characteristics of samples and area of sampling

Methodology	Study group	Area	Reference
Case-control	Adults	Riyadh	Almas et al. [17]
Case-control	Women	Riyadh	Awartani [18]
Case-control	Children	Riyadh	Wyne [19]
Case-control	Women	Hail	Farshori et al. [20]
Descriptive retrospective	Women	Al Madinah Al Munawwarah	Dar-Odeh et al. [21]
Cross-sectional	Adults	Saudi's Eastern Province	Madi et al. [22]
Cross-sectional	Adults	Riyadh	Alawaji et al. [23]
Case-control	Children	Riyadh	AlMutairi et al. [24]
Cross-sectional	Adults	Abha, Saudi Arabia	Alahmari et al. [25]

**Table 2:** Characteristics of study participants including sample size, age, gender, and diabetic and periodontal disease frequency in included studies T1DM: type 1 diabetes mellitus, T2DM: type 2 diabetes mellitus, HbA1c: glycated hemoglobin, SD: standard deviation.

Reference	Sample size	Type	Gender	Gingivitis/periodontitis	Mean age ± SD
Almas et al. [17]	40 (20 diabetic, 20 nondiabetic)	T2DM	Both genders break up not provided	Not mentioned	Not mentioned
Awartani [18]	126 (Group I (HbA1c <9%) 74, Group II (HbA1c >9%) 52)	T2DM	Female 126, male 0	Group 1 (7.3%), Group II (15.9%)	Group I (42.2 ± 6.7), Group II (45.7 ± 7.4)
Wyne [19]	321 (134 diabetics, 177 nondiabetics)	T1DM	Diabetic: 54 males and 80 females, nondiabetic: 86 males and 91 females	Gingivitis: diabetic 72.4%, healthy 60%	Diabetic 9.13 years old, healthy 7.87 years old
Farshori et al. [20]	318 (204 diabetics, 114 nondiabetics)	Type I 29.1% and type II 70.9%	Female 318, male 0	Nondiabetic (gingivitis, 89%, periodontitis, 31%). Diabetic (gingivitis, 95%, periodontitis 65%)	Diabetic 48.3 years old, healthy 33.82 years old
Dar-Odeh et al. [21]	1768(66 diabetic)	T2DM	Female 1768, male 0	Not mentioned	31.2 ± 10.13 years
Madi et al. [22]	190 (diabetic 62)	T2DM	Female 134, male 56	Tooth loss 156	Not mentioned
Alawaji et al. [23]	431 (diabetic 63)	T2DM	Female 286, male 183	Periodontal disease 384	35.4±13.3
AlMutairi et al. [24]	80 (40 diabetic, 40 nondiabetic)	T1DM	Diabetic: 24 males and 16 females. Nondiabetic: 24 males and 16 females.	Gingival index (diabetic 8.28, nondiabetic 6.88)	13.27 ± 1.09
Alahmari et al. [25]	499	T2DM	Female 343, male 156	Periodontitis 174	Not mentioned

Three of the five case-control studies included adult patients with and without diabetes. Most studies included only female patients. Patients with T1DM and T2DM were equally represented. Blood glucose levels and PDs were directly related to each other. Studies found a relationship between poorly controlled blood sugar levels and increased incidences of periodontitis among diabetic individuals. The diabetic individuals exhibited more severe PD, gingivitis, higher blood glucose levels, and less frequent tooth brushing or maintenance of healthy oral hygiene compared to healthy individuals. Additionally, conditions such as tooth loss, dental caries, retained roots, and other systemic diseases like hypertension are also linked to diabetes [17, 18, 20].

One study found that among nondiabetic patients, the incidence of periodontitis was only 10%, compared to 40% among diabetic patients. Moreover, diabetic patients had higher mean Community Periodontal Index of Treatment Needs (CPITN) scores and more missing teeth than healthy controls [17]. Another study's findings indicated that poorly controlled diabetic patients had a higher percentage of calluses and an increased risk of periodontitis. The study also highlighted a significant correlation between these patients' attachment loss (3-4 mm) and PD [18]. Furthermore, diabetic individuals experienced a statistically significant increase in tooth loss, particularly of premolars and molars, in both groups [20].

Two case-control studies that included children with T1DM reported opposite findings. Children with T1DM had better oral health compared to healthy children [19, 24]. Children with IDDM exhibited better oral health regarding dental caries and gingival status than their age-matched controls. However, they had significantly lower scores in oral health domains, suggesting poorer overall oral health-related quality of life (OHRQOL) in children with IDDM [24]. However, one study reported that a significant percentage of diabetic children had mild to moderate gingivitis. For children with diabetes, the mean DMFT (decayed, missing, and filled teeth) scores were 4.87±3.97 compared to 7.17±4.74 for healthy children. Additionally, a vast majority (83.6%) of the children with diabetes had fair or poor dental hygiene [19].

The cross-sectional studies reported that individuals with periodontitis who also have diabetes and other comorbidities are more likely to develop alveolar bone loss, independent of gender or smoking status [21, 22]. The degree of PD was significantly correlated with DM. Patients with DM had a higher risk of clinical attachment loss (CAL) compared to those without DM. The risk was also significantly higher in individuals with both DM and hypertension (HTN) [21, 22, 23]. According to CDC/AAP guidelines, there is a correlation between uncontrolled DM and periodontitis [23]. Another study revealed a prevalence of 7.4% among T1DM patients, 46.4% among T2DM patients, and an overall prevalence of 34.9% among both groups. The results indicated a higher incidence of periodontitis among T2DM patients. Most patients had severe periodontitis, and periodontitis was 12.7 times more common in people with T1DM over 37 years old [25].

Different studies reported various risk factors. For instance, one study reported that risk factors included being 35 years of age or older, being male, having a lower income, having uncontrolled DM, and currently smoking cigarettes, all of which were linked to untreated periodontitis [25]. Age was also found to be a risk factor for periodontitis, along with smoking and diabetes [25]. Other risk factors for the incidence of PDs, including smoking, were found to be major contributors to gum disease. Women are more susceptible to oral diseases like dental caries and periodontitis due to frequent hormonal changes at different stages of life (puberty, menstruation, pregnancy, and menopause). Poorly maintained oral hygiene, especially during pregnancy, can exacerbate disease [20].

The authors also highlighted other factors that are not directly related to PD in patients with diabetes but contributed to delays in seeking medical care. These included the complexity of their medical and dental history, compounded by language barriers, low education, financial constraints, and limited access to healthcare [21].

Risk of Bias Assessment

**Figure 2:**
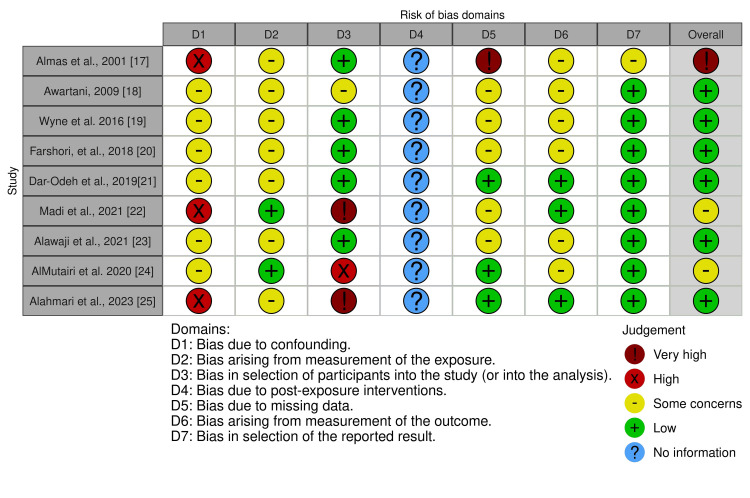
The risk of bias analysis of selected studies

The risk of bias analysis was conducted to assess potential biases in the studies across specific domains. The results are presented in Figure 2. Three studies [17, 22, 25] showed a high risk of bias due to confounding, whereas the remaining studies showed some concerns. Regarding "bias arising from the measurement of the exposure," two out of nine studies showed low bias, while the remaining studies showed "some concerns." In the domain of "bias in the selection of participants into the study," five studies revealed low bias, but two studies were at very high risk of bias. Three studies reported missing data; one of these showed a very high risk of bias, while the remaining two revealed some concerns. Almost all studies showed low to some concerns regarding bias in the measurement of the outcome and bias in the selection of the reported result. Overall, six studies showed a low risk of bias, two showed some concerns, and one was assessed as having a very high risk of bias.

Discussion

This systematic review evaluated the relationship between PDs and DM in the Saudi population. After an extensive literature search and applying inclusion and exclusion criteria, nine studies were selected for the systematic review. The studies highlighted the bidirectional nature of DM and PDs, with most focusing on the impact of diabetes on periodontal health.

Studies by Wyne [19] and AlMutairi et al. [24] found no significant difference in the oral health of diabetic and nondiabetic children. Both healthy and diabetic children demonstrated poor to moderate oral hygiene routines. These studies align with results already published worldwide, including in Saudi Arabia [26]. Interestingly, Wyne found that while diabetic children had a lower prevalence of caries compared to healthy children, they exhibited higher rates of gingivitis. This suggests that the impact of diabetes on oral health may vary across different age groups and types of dental conditions. There is ongoing debate concerning the possibility of dental caries in type 1 diabetes. According to another study, children with type 1 diabetes had a greater incidence of dental caries than the control groups [27]. Dental deterioration in children with diabetes has been linked to poor oral hygiene [28], and reduced salivary pH and flow rate have been identified as contributing factors in patients with T1DM [29]. Furthermore, studies linked a diet low in refined carbohydrates, which is less cariogenic, to the reduced caries rates seen in children with managed diabetes [30, 31].

Out of the nine selected studies, three focused on the adult population with T2DM in both men and women. Almas et al. [17], Alawaji et al. [23], and Alahmari et al. [25] provided insights into the broader Saudi population, highlighting that T2DM is associated with increased PD severity. The studies found that diabetes, especially when poorly controlled, exacerbated periodontal conditions, contributing to higher clinical attachment loss and bone loss. The remaining studies, which focused on women only, reported similar results. The severity of both diseases can be heightened in females due to hormonal imbalances and other factors [32]. Many studies have reported a higher incidence of PD with gestational diabetes [33]. In the United Arab Emirates, a significantly greater percentage of patients with diabetes (23%) had a CAL ≥3mm than the nondiabetic control group (10%) (p = 0.01) [34].

According to multiple studies, people who struggle to regulate their blood glucose levels or who have neglected their diabetes are 2-3 times more likely to develop periodontitis, with the key determinant of risk being the patient’s degree of glycemic control [35]. Furthermore, long-term studies have shown that people with diabetes have a higher frequency of progressing periodontitis. For instance, cross-sectional epidemiologic studies have demonstrated that people with periodontitis experience more severe and widespread loss of periodontal tissue associated with diabetes [36]. Effective periodontal therapy has been shown to lower circulating levels of C-reactive protein (CRP) and tumor necrosis factor (TNF) in diabetic individuals, indicating its direct involvement in reducing inflammation, according to several carefully monitored human investigations [37].

The studies were limited by small sample sizes and data unavailability for other factors that might have influenced the diseases. The limitations and outcomes of the study are detailed in Table 3. This study is the first to be conducted exclusively on data regarding diabetes and PDs in Saudi Arabia. The study finds that there are very few reports with very small sample sizes on the impact of type 1 and type 2 DM on oral health in the Saudi population.

**Table 3:** The outcomes and limitations of selected studies PD: periodontal disease, CPITN: community periodontal index of treatment needs, DM: diabetes mellitus, CDC: Centers for Disease Control and Prevention, AAP: American Academy of Periodontology, OHRQoL: oral health-related quality of life.

Reference	Outcome	Limitations
Almas et al. [17]	Oral hygiene was found to be the most important factor in the development of PD as nonregular brushers have a higher incidence of the disease. Diabetic patients had higher mean CPITN scores, and more missing teeth compared to healthy subjects	Very small sample size; No other parameters are mentioned. The study did not analyze the effect of other possible confounding factors such as diet, smoking, and medication use, which could influence the severity of periodontitis
Awartani [18]	The patient in the poor glycemic control group had more sites with PD ≥4 mm, and the percentage of sites with 3–4 mm attachment loss was significantly higher in group II than in group I. More sites of 1–2 mm attachment loss were observed in group I than in group II	Small sample size
Wyne [19]	The prevalence of caries and severity of PD were high in both type 1 diabetic children and healthy controls. However, overall caries experience was lower in diabetic children than the healthy children. The level of gingival health was similar in diabetic and healthy children	Only focused on children
Farshori et al. [20]	The study showed a statistically significant increase in tooth loss among diabetics compared to the control group	study analyzed only the female population selection bias may be present in the study since the samples for diabetic and nondiabetic groups were selected from different populations, which could have affected the results. The study did not analyze the effect of other possible confounding factors such as diet, smoking, and medication use, which could influence the severity of periodontitis in female populations.
Dar-Odeh et al. [21]	The study found that DM and Hypertension were independently associated with a higher experience of missing teeth. However, the study did not report a significant association specifically between various teeth and systemic conditions, such as DM and hypertension	It was a retrospective analysis of patients' clinical records. There was no data on oral hygiene practices or sociodemographic factors that may influence oral health
Madi et al. [22]	The study found a significant association between periodontal disease and comorbidities such as diabetes, hypertension, hyperlipidemia, and coronary heart disease	Small sample size
Alawaji et al. [23]	The article mentions an association between uncontrolled diabetes mellitus and periodontitis in the unadjusted models; however, the adjusted association was significant only with CDC/AAP severe periodontitis. This finding may indicate that the risk association with diabetes mellitus is more pronounced at a more severe periodontitis threshold	The results have limited external validity and have selection bias since it targeted a specific sample of untreated subjects who were recruited from screening dental school clinics
AlMutairi et al. [24]	The authors found a correlation between oral health and different domains of OHRQoL in children with type-1 diabetes mellitus	Small sample size
Alahmari et al. [25]	overall prevalence of periodontitis among DM patients was 34.9%, ranging from 7.4% among type I DM patients to 46.4% among type II DM patients. Moreover, age and smoking were the potential factors associated with periodontitis prevalence among type I and II DM	A convenience sample prevented some of the variables from being significant. A nonrandom selection of the study participants might affect the representation of the participants. Missing data on confounding factors.

## Conclusions

This systematic review highlights a significant association between DM and PD in the Saudi population, although mixed results regarding the strength of this association with various PDs were observed. Most reports focus only on women and children; no comprehensive report exists that includes data from men, women, and children while considering all related comorbidities and confounding factors. The findings collectively reinforce the established notion that diabetes and PD have a reciprocal relationship, with each condition potentially exacerbating the other.

Future research should aim to conduct longitudinal studies to better understand the causal relationships between diabetes and PD. Larger, multicenter studies in Saudi Arabia, using standardized diagnostic criteria for both conditions, are needed to improve the reliability and comparability of findings. Additionally, exploring the biological mechanisms linking diabetes and PD could lead to better-integrated treatment approaches. Investigating the impact of targeted interventions, such as improved glycemic control and comprehensive periodontal therapy, on the progression and management of both conditions could provide valuable insights into effective treatment strategies.
